# Probing the Effect of Two Heterozygous Mutations in Codon 723 of *SLC26A4* on Deafness Phenotype Based on Molecular Dynamics Simulations

**DOI:** 10.1038/srep10831

**Published:** 2015-06-02

**Authors:** Jun Yao, Xuli Qian, Jingxiao Bao, Qinjun Wei, Yajie Lu, Heng Zheng, Xin Cao, Guangqian Xing

**Affiliations:** 1 Department of Biotechnology, School of Basic Medical Science, Nanjing Medical University, Nanjing, P.R. China; 2 School of Life Science and Technology, China Pharmaceutical University, Nanjing, P.R. China; 3 Department of Otolaryngology, the First Affiliated Hospital of Nanjing Medical University, Nanjing, P.R. China

## Abstract

A Chinese family was identified with clinical features of enlarged vestibular aqueduct syndrome (EVAS). The mutational analysis showed that the proband (III-2) had EVAS with bilateral sensorineural hearing loss and carried a rare compound heterozygous mutation of *SLC26A4* (IVS7-2A>G, c.2167C>G), which was inherited from the same mutant alleles of IVS7-2A>G heterozygous father and c.2167C>G heterozygous mother. Compared with another confirmed pathogenic biallelic mutation in *SLC26A4* (IVS7-2A>G, c.2168A>G), these two biallelic mutations shared one common mutant allele and the same codon of the other mutant allele, but led to different changes of amino acid (p.H723D, p.H723R) and both resulted in the deafness phenotype. Structure-modeling indicated that these two mutant alleles changed the shape of pendrin protein encoded by *SLC26A4* with increasing randomness in conformation, and might impair pendrin’s ability as an anion transporter. The molecular dynamics simulations also revealed that the stability of mutant pendrins was reduced with increased flexibility of backbone atoms, which was consistent with the structure-modeling results. These evidences indicated that codon 723 was a hot-spot region in *SLC26A4* with a significant impact on the structure and function of pendrin, and acted as one of the genetic factors responsible for the development of hearing loss.

Hearing loss is one of the common sensorial disorders with an incidence of approximately 1 in 1000 children worldwide. At least half of these cases are attributed to genetic factors and present different types of hearing loss (syndromic and nonsyndromic). About 80% of these genetic-causative cases were classified as nonsyndromic hearing loss (NSHL)^1^. Many genes have been described for NSHL in autosomal recessive (ARNSHL), autosomal dominant (ADNSHL), X-linked and maternal inheritance patterns. ARNSHL accounts for 60-70% of inherited NSHL cases^1^,^2^,^3^. To date, about 100 loci for ARNSHL (DFNB) have been mapped in the human genome and over 50 genes have been identified (http://hereditaryhearingloss.org/).

Enlarged vestibular aqueduct (EVA) is a common form of inner ear abnormality and clinically characterized by disequilibrium and fluctuating or progressive sensorineural hearing loss^4^. EVA could occur in DFNB4 cases designated as EVA syndrome (EVAS) but with no thyroid anomalies, or in the cases of Pendred syndrome (PS) featured by cochlear abnormalities, congenital sensorineural hearing loss, goiter and positive perchlorate discharge test^5^,^6^. Recessive mutations in the anion transporter gene *SLC26A4* (OMIM 605646) is considered to be the main genetic cause of DFNB4 and PS. *SLC26A4*, located in the same genomic region (7q22-31) allelic to the *PDS* gene, encodes a highly hydrophobic membrane protein named pendrin (NP_000432.1), which is a member of the *SLC26* family of anion transporters and expressed in the cochlea, thyroid, kidney, and placenta^7^,^8^. In the inner ear, pendrin is found in the endolymphatic duct and sac, where it functions as a chloride/iodide and bicarbonate exchanger and plays a role in inner ear fluid homeostasis^9^,^10^.

*SLC26A4* has an extensive mutation spectrum spreading over all the exons and their flanking sequences, the majority of them are missense mutations, frameshifts, insertions or deletions, and aberrant splice site alterations^11^. PS patients were always detected with the biallelic mutations in *SLC26A4* consistent with autosomal recessive disorder, and no biallelic mutations were found in EVA-negative patients^5^. Up to now, more than 200 *SLC26A4* mutations of have been described (www.healthcare.uiowa.edu/labs/pendredandbor) and the mutation spectrum varies among different ethnic groups^5^,^12^,^13^,^14^,^15^,^16^,^17^,^18^,^19^,^20^,^21^,^22^,^23^,^24^,^25^. In Asian populations, a high frequency of detecting *SLC26A4* mutations was reported in EVAS patients, and a large proportion of these cases were identified with a form of biallelic mutants. IVS7-2A>G and c.2168A>G (p.H723R) were the two most prevalent mutant alleles in *SLC26A4*^13^,^14^,^19^,^20^,^21^,^22^,^23^,^24^,^25^. *SLC26A4* mutations are the second-most common cause of deafness in China^22^,^23^,^24^,^25^, and there was 88.4% of EVA-positive patients detected with biallelic variants of *SLC26A4*, 9.5% with monoallelic mutations and 2.1% with no mutant alleles, and IVS7-2A>G mutation was identified as the most common form accounting for 57.63% of the mutant alleles^19^,^20^. Furthermore, a significant phenotypic heterogeneity was observed among PS and DFNB4 patients, which might be attributed to environmental or genetic factors. Thus, the molecular etiology of hearing loss associated with *SLC26A4* mutations, as well as the genotype-phenotype correlation, was still needed to be further investigated.

In this study, a rare compound heterogeneous mutation of *SLC26A4* (IVS7-2A>G, c.2167C>G) was identified in a Chinese family with EVAS, while another confirmed pathogenic biallelic mutation in *SLC26A4* (IVS7-2A>G, c.2168A>G) was also detected in our work. Interestingly, these two biallelic mutations in *SLC26A4* shared one common mutant allele (IVS7-2A>G) and the same codon of the other mutant allele (p.H723D, p.H723R), but led to different changes of amino acid and both resulted in the deafness phenotype, which indicated that codon 723 in *SLC26A4* could be a hot-region responsible for the development of hearing loss. To elucidate the molecular etiology of hearing loss associated with codon 723 in *SLC26A4*, we conducted an in silico analysis, including structure-modeling and molecular dynamics simulation (MDS) upon above two mutations to analyze the probable loss-of-function mechanisms of pendrin mutants associated with the deafness phenotype.

## Results

### Patients and clinical investigation

**Figure 1:**
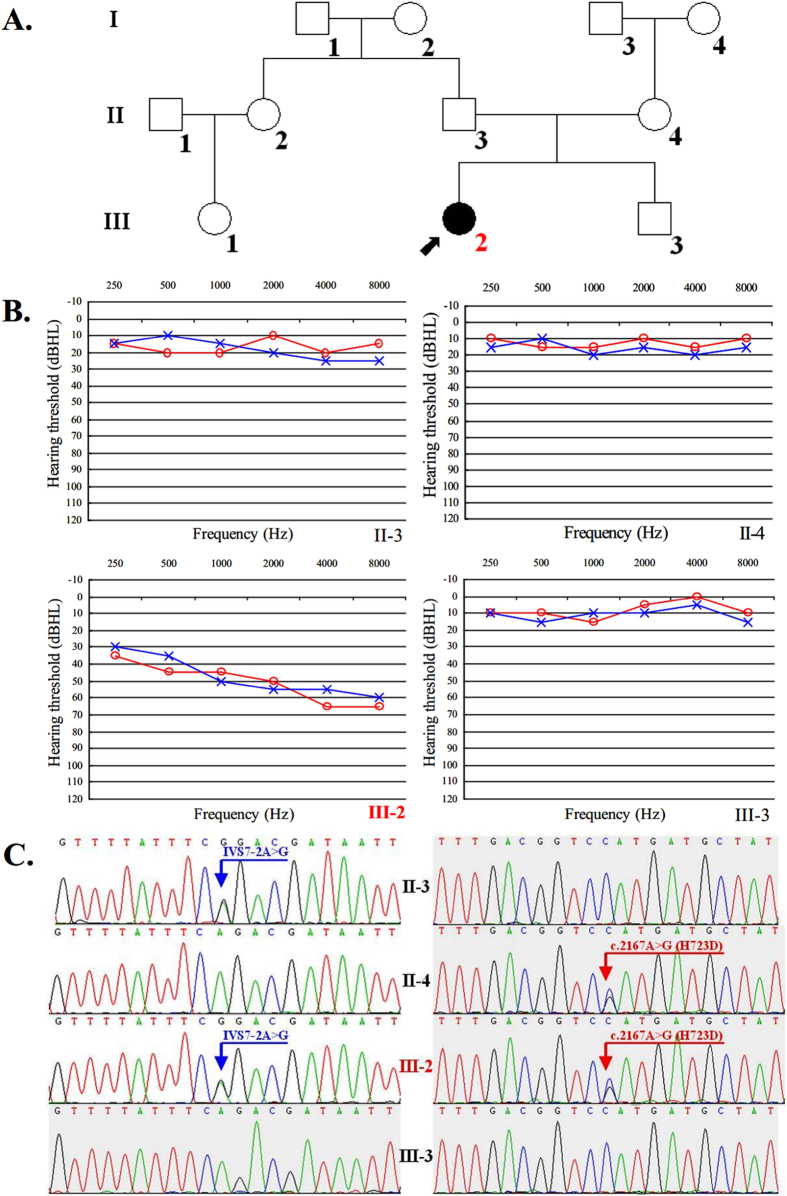
Pedigree of an affected Chinese family with EVAS (**A**) (Filled symbols represent affected individuals of males (squares) and females (circles), and empty, unaffected ones. An arrow denotes the proband), audiological evaluation (**B**) and mutational analysis of partial family members (**C**).

The proband (III-2) was derived from a Chinese family spanning 3 generations (Fig. 1A). The parents (II-3, II-4) presented normal hearing. Comprehensive examinations of the family medical history did not identify any other clinical syndromic features. No pre-, peri- or postnatal risk factors for hearing loss were identified. PTA test showed that the proband (III-2) exhibited moderate bilateral sensorineural hearing loss (Fig. 1B). The otoscopy showed normal external auditory canal and tympanic membranes. Other audiological examinations, including immittance, ABR, and DPOAEs were also performed and suggested a cochlear involvement. Temporal bone CT scan showed normal middle ear structure and internal auditory canal, but a common form of inner ear bony malformation, EVA, was detected. No symptom of goiter was observed and the thyroid function was normal.

### Mutational analysis of SLC26A4

**Figure 2:**
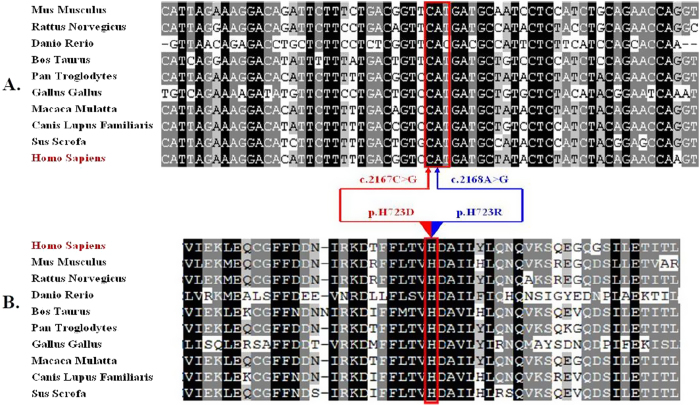
Multiple alignment of *SLC26A4* (**A**) and its amino acid sequence (**B**). The conservation analysis shows that p.H723D (c.2167C>G) (red arrow) and p.H723R (c.2168A>G) (blue arrow) heterozygous missense mutations in *SLC26A4* are both at a highly conserved position by comparison to the corresponding sequence of Homo Sapiens, Rattus Norvegicus, Danio Rerio, Bos Taurus, Pan Troglodytes, Gallus Gallus, Macaca Mulatta, Canis Lupus Familiaris, Sus Scrofa.

The above clinical investigations suggested that the proband (III-2) suffered from a disease of EVAS. The mutational analysis indicated that the proband (III-2) carried a compound heterozygote of two mutant alleles (IVS7-2A>G, c.2167C>G) in *SLC26A4* with negative mutations of other NSHL-causative genes. Further genetic analysis for the proband’s parents revealed that the father (II-3) was a heterozygous carrier of the IVS7-2A>G mutation in the splice site of intron 7, and her mother carried a heterozygote of the c.2167C>G mutation in exon 19 (Fig. 1C). None of any *SLC26*A4 mutations was detected in the brother (III-3). The genotypes of *SLC26A4* seemed to be responsible for the phenotypes of the proband (III-2) and her parents (II-3, II-4). It was well-known that c.2168A>G (p.H723R) was one of the prevalent mutations of *SLC26A4* in Asian populations^13^,^14^,^19^,^20^,^21^,^22^,^23^,^24^,^25^. Similar to the mutation of c.2168A>G (p.H723R), the mutation of c.2167C>G (p.H723D) is most likely pathogenic due to its evolutionary conservation and conserved amino acid change (Fig. 2). The functional impact of c.2167C>G (p.H723D) on the protein was also assessed using SIFT (http://sift.jcvi.org) and PolyPhen-2 (http://genetics.bwh.harvard.edu/pph2/), and this mutation was predicted to be functional deleterious. It was noteworthy that these two mutations both presented deafness phenotypes.

We then performed further genetic analysis on the mutation of c.2167C>G. Twenty-five probands with ARNSHL (obtained from Otology Clinic of the First Affiliated Hospital of Nanjing Medical University), 150 sporadic patients (obtained from Nanjing City School for Deaf Children) and 200 race matched controls with normal hearing were subjected to Sanger sequencing and mutational analysis of *SLC26A4* in intron 7 and exon 19. As a result, no patients or controls were detected with the mutation of c.2167A>G, and two sporadic patients with bilateral hearing loss were identified with a reported compound heterozygous mutation of *SLC26A4* (IVS7-2A>G c.2168A>G), meanwhile no monoallelic or biallelic mutations of c.2167C>G in *SLC26A4* were identified in these participants.

### Model building and structure-based analysis

**Figure 3:**
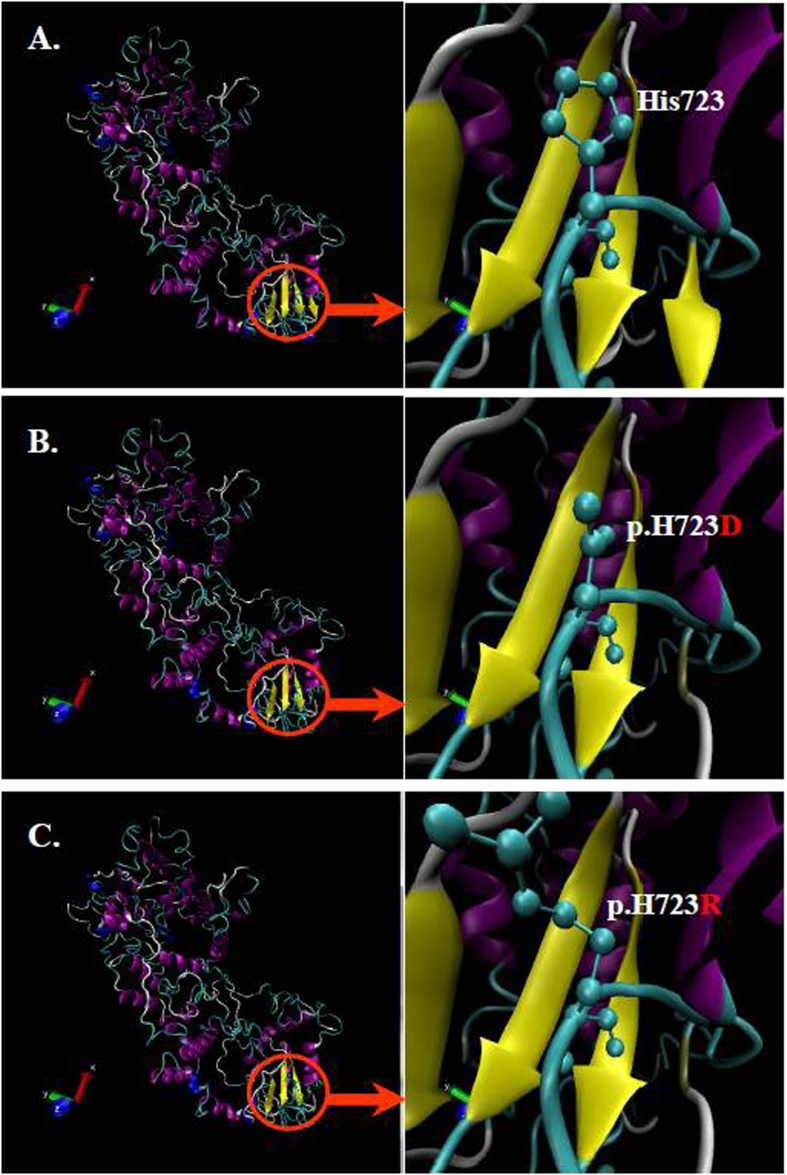
The structure modeling of wild-type pendrin (**A**), p.H723D mutant (**B**) and p.H723R mutant (**C**). The mutated site was emphasized by a red circle and locally zoomed.

As mentioned above, two compound heterozygous mutations of *SLC26A4* (IVS7-2A>G/c.2167C>G, IVS7-2A>G/c.2168A>G) were detected in EVAS patients. Considering a common mutant allele (IVS7-2A>G) was shared by these two mutations and located in the splice site, the structure-based analysis was focused on the other mutant allele (c.2167C>G or c.2168A>G) in coding sequence (CDS) of *SLC26A4*. For the submitted amino acid sequence of pendrin, the three-dimension structure of pendrin was modeled based on the lowest energy using Robetta online program (http://robetta.bakerlab.org/). In order to build the mutant structures, we made a point mutation in wild-type pendrin protein at p.H723D (c.2167C>G) and p.H723R (c.2168A>G) respectively, and the mutant structures were built using the above modeled pendrin as a template (Fig. 3).

Pendrin, encoded by *SLC26A4* cDNA sequence, was identified to have 12 transmembrane (TM) domains with both the amino (NH_2_) and carboxyl (COOH) terminus residing intracellularly^7^,^26^,^27^. The mutations of c.2167C>G (p.H723D) and c.2168A>G (p.H723R) were both located at the same codon in the coding region of carboxyl terminus residues (codon 504 to 780). The structural modeling also revealed that these two mutations (Fig. 3B,C) altered the structure of pendrin protein and resulted in increased randomness in conformation, which may impair pendrin’s ability as an anion transporter. It was strongly suggested that these two mutations in codon 723 could be functionally deleterious.

### Molecular dynamics analysis

To examine the results obtained from structure modeling and the possible functional impacts of the two mutations (p.H723R and p.H723D) on the deafness phenotype, we further conducted molecular dynamics simulation (MDS) for wild-type and mutant pendrin proteins. The variation of root mean square deviation (RMSD) and root mean square fluctuation (RMSF) between wild-type and mutant structures were evaluated. As shown in Fig. 3, the modeled pendrin protein was predicted to mainly comprise domain I (codon 1-514) and domain II (codon 515-780). The mutant positions were both located in Domain II, which was just consistent with the region of carboxyl terminus (COOH) of pendrin. Therefore, the MDS was conducted focusing on the changes of domain II and proceeded as followed: initially the backbone atoms in domain I and II were restrained with a harmonic force of 2.0 kcal/(mol Å^2^) and subjected to MDS for 10 ns, and then only the backbone atoms in domain I was restrain with a harmonic force of 0.2 kcal/(mol Å^2^) and subjected to MDS for 20 ns. MDS was conducted as mentioned in section 2.4. In addition, the region from Gly740 to the end of carboxyl terminus was excluded in MDS, due to its irregular second structures in both of wild-type and mutant pendrins.

**Figure 4:**
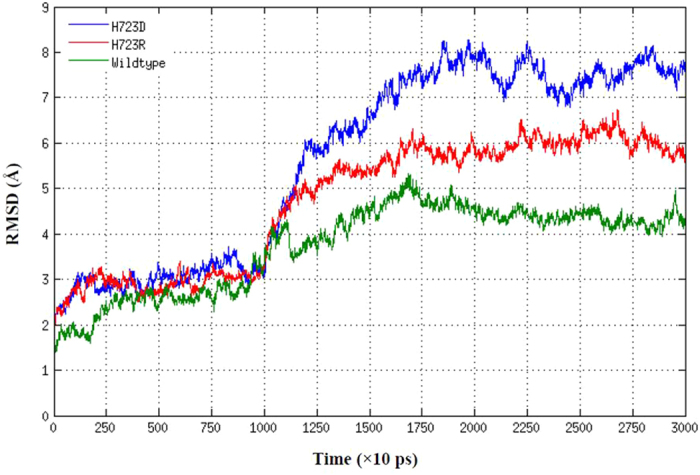
Backbone (from Phe515 to Gly740) RMSDs are shown as a function of time for wild-type pendrin (in green), p.H723D mutant (in blue) and p.H723R mutant (in red) protein structures at 310 K.

The relative structural drift or stability of pendrin structure was measured as RMSD values for the Cα atoms from the initial structures. As shown in Fig. 4, at the first 10 ns of simulation, the RMSD were relatively stable for both wild-type and mutant pendrins, because the backbone atoms of the proteins were restrained. After removing the restrain at 10 ns, the RMSD increased rapidly. Compared with wild-type pendrin system, the RMSD values of mutant system increased more significantly, indicating that mutant-type pendrins underwent a larger conformational change than wild-type pendrin, which was consistent with the presumption that the less of secondary structure (*α*-helix and *β*-sheet) observed in mutants may cause higher structural flexibility. The RMSD of all systems reached to equilibrium after 20–30 ns of simulations, and the trajectories with stabilized RMSD were subjected to the following analysis of second structures and stability evaluation.

**Figure 5:**
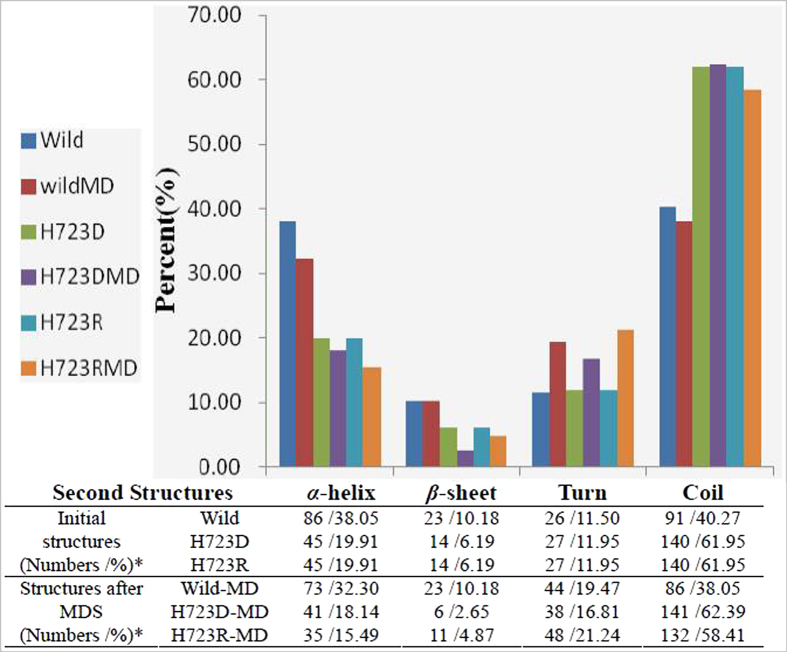
The analysis of second structures in wild-type and mutant pendrins before and after MDS (* present the residue numbers of amino acid /percentage % in second structures).

The second structures in wild-type and mutant pendrins were analyzed before and after MDS (Fig. 5). In both initial and MD structures, less *α*-helixes/*β*-sheets and more coils were present in mutants as compared with wild-type pendrin. It was noted that the percentage of *β*-sheet in wild-type pendrin remained invariant before and after MDS, while the *α*-helix and *β*-sheet in mutants were decreased after MDS, especially, the percentages of *β*-sheet in p.H723D and *α*-helix in p.H723R were observed significantly reduced. These results indicated that mutation at position 723 may influence the stability of the pendrin, and result in improperly folding of the protein, thus disturbing its function.

**Figure 6:**
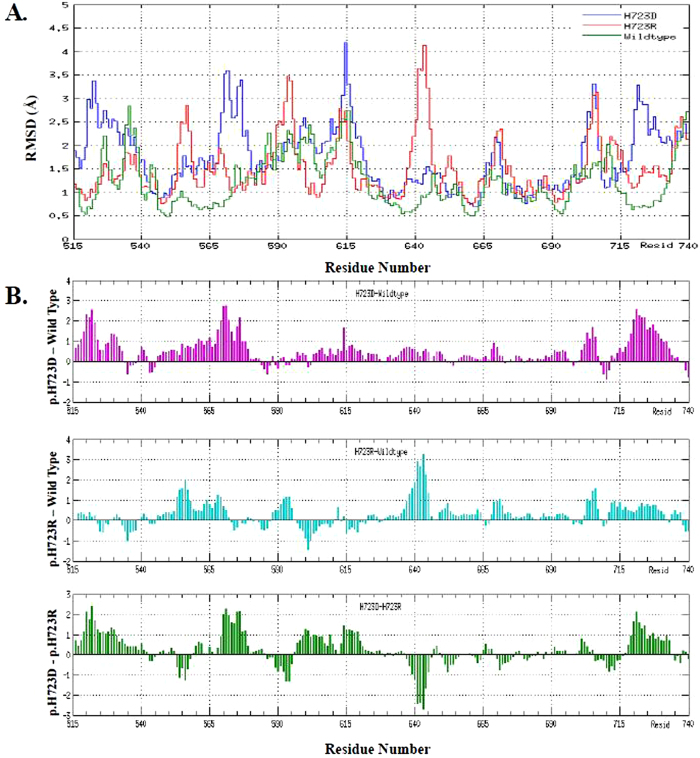
RMSF (**A**) of the backbone Cα atoms (from Phe515 to Gly740) of wild-type pendrin (in green), p.H723D mutant (in blue) and p.H723R mutant (in red) versus time at 310 K, and their comparative RMSF (**B**): p.H723D-Wild type, p.H723R-Wild type, p.H723D-p.H723R.

To determining whether the mutations affected the dynamic behavior of residues, the RMSF values of wild-type and mutant pendrins’ backbone residues (from Phe515 to Gly740) were calculated with respect to the starting structures (Fig. 6A). Analysis of fluctuation score showed that low degree of flexibility was presented in the wild-type system as compared with the mutants’ structure. Additionally, in mutant systems, more residues of p.H723D mutant seemed to have high values of RMSF than that of p.H723R mutant (Fig. 6B). The above suggested that the stability disparity of domain II might be responsible for functional variation among wild-type, p.H723R and p.H723D mutants of pendrin.

## Discussion

In this study, we reported an affected family consistent with autosomal recessive disorder caused by bi-allelic function loss of pendrin protein. The proband (III-2) had EVAS with moderate bilateral sensorineural hearing loss and carried a rare compound heterozygous mutation of *SLC26A4* (IVS7-2A>G, c.2167C>G), which was inherited from the same mutant alleles of IVS7-2A>G heterozygous father (II-3) and c.2167C>G heterozygous mother (II-4), who both had normal hearing. The c.2167C>G mutation of *SLC26A4* was firstly reported in an Inner Mongolia sporadic patient carrying a compound heterozygous mutation of c.2167C>G and c.2168A>G^20^, and it was infrequent to be detected in EVAS or PS affected families until it was re-identified in our present work.

As was known that the IVS7-2A>G mutation was the most prevalent *SLC26A4* mutation among Asian populations, such as Chinese, Japanese and Korean, but rarely detected in the Western populations^5^,^12^,^13^,^14^,^15^,^16^,^17^,^18^,^19^,^20^,^21^,^22^,^23^,^24^,^25^. Although the effect of the IVS7-2A>G mutation on pendrin loss-of-function had been proved that it changed the conserved nucleotide of the acceptor splice site and caused the deletion of entire exon 8, resulting in a truncated protein consisting of only 310 amino acids^28^, the pathogenic potential and phenotypic variation of this mutation remained uncertain. Patients carrying the heterozygous mutation of IVS7-2A>G could develop hearing loss in the presence of additional genes (mutant allele) or environmental factors. However, in inherited family cases, it was highly possible that the IVS7-2A>G mutation could synergistically function with other inherited nucleotide changes and represent a common or uncommon polymorphism resulting in different phenotypes.

Besides the compound heterogynous mutation of IVS7-2A>G and c.2167C>G identified in this study, another common compound heterozygous mutation of IVS7-2A>G and c.2168A>G was also detected in our previous and present work (data not shown). The mutant allele of c.2168A>G (H723R) has been reported to be another common pathogenic mutation in Asian populations^5^,^12^,^13^,^14^,^15^,^16^,^17^,^18^,^19^,^20^,^21^,^22^,^23^,^24^,^25^. Patients with one of these two biallelic mutations were suffering from EVAS with bilateral sensorineural hearing loss. Considering these two biallelic mutations shared one common mutant allele (IVS7-2A>G) and their other mutant alleles were both located at the same codon (codon 723) in the coding region of carboxyl terminus of pendrin, it was presumed that the genotype of their other mutant allele (c.2168A>G or c.2167C>G) could lead to a different conserved amino acid change (p.H723R or p.H723D), but both cause a deleterious impact on the protein function of pendrin, and eventually result in pathogenicity with deafness phenotypes.

It has been suggested that the defect in chloride/iodide and bicarbonate exchange activities of pendrin at the apical membrane of inner ear epithelial cells is the key factor that causes EVA and deafness^29^. Taylor *et al.*^27^ and Rotman-Pikielny *et al.*^30^ investigated the effect of *SLC26A4* missense mutations on pendrin localization and iodide transport in mammalian cell lines. Their results indicated that most of *SLC26A4* mutants appeared to be retained within the endoplasmic reticulum (ER) following transfection, and loss of pendrin iodide transport was observed for all mislocalizing mutations^27^,^30^. It was also found that p.H723R mutant was mostly expressed in ER of p.H723R-transfected cells, and the lack of anion exchange activity was also detected^31^,^32^. Via functional tests of mutated pendrin allelic variants using the heterologous expression system, Yuan *et al.*^23^ also revealed that the pathology of *SLC26A4* mutations could be linked to reduction or loss-of-function in the ion transport activity of pendrin, and it is noted that four of the ten tested *SLC26A4* variants [c.1517T>G (p.L506R), c.1985G>A (p.C662Y), c.1991C>T (p.A664V), c.2326C>G (p.R776G)] found in PS and EVAS patients are located in carboxyl terminus region of pendrin. In fact, there are at least 40 mutations of *SLC26A4*, including c.2168A>G (p.H723R) and c.2167C>G (p.H723D), were identified in this region (www.healthcare.uiowa.edu/labs/pendredandbor), which indicated that the carboxyl terminus could be an important intracellular part of pendrin in function of an anion transporter.

However, genotypic and phenotypic heterogeneity still made the diagnosis of PS or EVAS uncertain and difficult to reach a consensus on the development of hearing loss, and the pathogenic potential of *SLC26A4* mutations and the mechanism of phenotypic heterogeneity still remained to be further elucidated. In our study, the identification of the c.2167C>G (p.H723D) mutation along with the c.2168A>G (p.H723R) mutation just offered us an opportunity to contrastively evaluate their structural and functional impacts on pendrin at the same codon, and possibly reveal the genotype-phenotype correlation for these two mutations. To clarify the molecular mechanism involved in conformational changes of protein, a combination of molecular dynamics theory and simulation approach was indispensible that could offer us informative data on thermodynamic and kinetic behavior at the atomic level, and help in associating the conformational changes with functional alterations as well as the varied phenotypes in disorders. We observed notable changes of mutant pendrins (p.H723R and p.H723D) as compared with wild-type pendrin by structure modeling and MDS. The MDS results also indicated that the stability of mutant pendrins was reduced and induced the increased flexibility of backbone atoms and randomness in conformation, which was consistent with the modeled structures of wild-type and mutant pendrins. Based on mentioned above, it was suggested that the aberrant structures and destabilization of the mutants could be the structural basis of subcellular mislocalization and malfunction of mutant pendrins.

In summary, we reported a rare compound heterozygous mutation of IVS7-2A>G and c.2167C>G in *SLC26A4* in an affected Chinese family with EVAS, and conducted a rudimentary investigation on the possible molecular explanation of hearing loss implicated with two allelic mutations (p.H723R and p.H723D) in codon 723 of *SLC26A4*. However, many other hot-spot regions in *SLC26A4* had been screened via a molecular epidemiological survey, due to the wide mutational spectrum of *SLC26A4* and its significant phenotypic heterogeneity, a further investigation on *SLC26A4* mutations associated with the functional reduction or loss of pendrin, as well as the development of hearing loss, will be carried out in our future work.

## Materials and Methods

### Patients and clinical investigations

A Chinese family associated with ARNSHL was described (Fig. 1) in this paper. The proband (III-2) (a girl of nine years old) suffered from pre-lingual hearing loss, while both of his parents and her brother (III-3) had normal auditory acuity. Patients or their parents were interviewed for a detailed medical history, including age at onset, evolution of hearing loss, family history, presence of tinnitus, medication, noise exposure, use of hearing aids, mother’s health condition during pregnancy and patient’s clinical history (infection, possible head or brain injury and the usage of aminoglycoside antibiotics). The hearing level was examined by pure-tone audiometry (PTA), immittance, auditory brainstem response (ABR), and distortion production otoacoustic emissions (DPOAEs). The audiological data was evaluated based on the criteria established by the European Working Group on Genetics of Hearing Loss. Temporal bone computerized tomography (CT) was performed for diagnosing EVA or inner ear malformation based on the criteria of a diameter of greater than 1.5 mm at the midpoint between the common crus and the external aperture^33^. The ultrasound scan (US) of thyroid, as well as the measurement of thyroid hormone, was conducted to evaluate goiter for PS. All these procedures were performed at the First Affiliated Hospital of Nanjing Medical University, Nanjing, China.

This study was performed according to a protocol approved by the ethics committee of Nanjing Medical University, Jiangsu province. Informed consent was obtained from the patients or their parents prior to blood sampling. All procedures used in this study conformed to the tenets of the Declaration of Helsinki. DNA samples were extracted from whole blood and used for mutational analysis.

### Mutational analysis

The mutational analysis was performed by PCR amplification and Sanger sequencing (ABI 3730, Foster City, USA) to detect the presence of mutations of NSHL-causative genes (including *GJB2*, mitochondrial DNA 12S rRNA and *SLC26A4* in intron 7 and exon 19). These coding sequences as well as exon-intron boundaries were sequenced and analyzed. Primers, flanking the coding exons (50–100 bp of the flanking intron regions), were designed using Primer Premier 5.0 software (Premier, Polo Alto, CA, USA) based on human genomic sequence and synthesized by BGI-Beijing, Shenzhen, China. Sequencing data was compared pair-wise with the human genome database, and the identified mutations were confirmed by the bidirectional sequencing. In addition, the mutational analysis was also conducted for the other members of this family (II-3, II-4, III-3). The possible pathogenic variant was evaluated according to the evolutionary conservation and change of conserved amino acid. SIFT (http://sift.jcvi.org) and PolyPhen-2 (http://genetics.bwh.harvard.edu/pph2/) were used to predict whether changes of amino acid affected protein function.

### Structure modeling of wild-type and mutant pendrins

The full length of *SLC26A4* cDNA encodes pendrin, a 780 amino acid residues protein with a molecular mass of 73 kDa. Due to the absence of pendrin and its homologues’ structures in Protein Data Bank, the three-dimension structure of pendrin was modeled by Robetta online program (http://robetta.bakerlab.org/). The structure of mutant pendrin was built by Swiss-Model Version 8.05 (http://swissmodel.expasy.org/). The wild-type and mutant pendrins’ structures were energetically optimized by applying the all atoms OPLS force field available under the GROMACS 4.5.3 package^34^, and C-terminal and N-terminal outgap modeling was conducted.

### Molecular dynamics analysis

Molecular dynamics simulation (MDS) was performed using Amber 12.0 and Ambertools 13.0 (University of California, San Francisco USA)^35^,^36^. The modeled structures of wild-type and mutant pendrins (p.H723R and p.H723D) were used for MDS. Systems were solvated in a periodic box with TIP3P water molecules at 8.0 Å from the protein atoms^37^. Counter-ions were added to neutralize the simulation systems. If not specified mentioned, ff12SB force field was used for systems during the simulation. To remove possible poor contacts between protein atoms and solvent, energy minimization (5000 steps for the water molecules followed by 10000 steps for the whole system) were performed before MDS. Langevin dynamics with the collision frequency 2 ps^−1^ was used to increase the temperature of the solvated system^38^, from 0 K to 310 K in 200 ps. At this stage, the backbone atoms of the protein were restrained by a harmonic force of 1.0 kcal/(mol Å^2^). Electrostatic interactions were calculated using the Particle Mesh Ewald method^39^, and the non-bonded cutoff was set to 10.0 Å. Next, each system was subject to two steps of MDS at 310 K under NPT ensemble conditions. At the first step, the backbone atoms in both domain I and II of the protein were restrained by a harmonic force of 2.0 kcal/(mol Å^2^) and subjected to 10 ns MDS. The second step, only backbone atoms in domain I was restrain by a harmonic force of 0.2 kcal/(mol Å^2^) , and then subjected to MDS for 20 ns. The time step was set as 2 fs during all MDS stage and a snapshot was saved every 10 ps. The PTRAJ analysis program within Ambertools 13.0 was used for the calculations of the root mean square deviation (RMSD) and the root mean square fluctuation (RMSF). The final structures of MDS refined pendrin and mutants were calculated as follows: the trajectories of the last 10 ns (from 20 ns to 30 ns) were clustered, and the biggest cluster was selected. An average structure was calculated from the cluster as the representing final model of MDS.

## Additional Information

**How to cite this article**: Yao, J. *et al.* Probing the Effect of Two Heterozygous Mutations in Codon 723 of *SLC26A4* on Deafness Phenotype Based on Molecular Dynamics Simulations. *Sci. Rep.* **5**, 10831; doi: 10.1038/srep10831 (2015).
